# DEMENTIA CARE COORDINATION FOR CAREGIVERS OF VETERANS DIAGNOSED WITH DEMENTIA: AN INNOVATIVE VA INITIATIVE

**DOI:** 10.1093/geroni/igac059.1967

**Published:** 2022-12-20

**Authors:** Judith Howe, Eve Gottesman, Nicholas Koufacos

**Affiliations:** Ichan School of Medicine at Mount Sinai, Bronx, New York, United States; James J. Peters VA Medical Center, Bronx, New York, United States; James J. Peters VA Medical Center, Bronx, New York, United States

## Abstract

There are expected to be 335,425 Veterans with dementia by 2033. Dementia has a significant impact on families, especially caregivers. Caregivers often report high levels of stress and burden, which can cause them to develop physical and mental illness, impacting the care they provide to family members with dementia. In 2021, under a Veterans Health Administration (VHA) directive, the Bronx VA Geriatric Research, Education and Clinical Center developed a Dementia Care Coordination program, funded by the VHA Office of Rural Health sponsored Geriatric Scholars Program. Led by a Dementia Care Coordinator (DCC), who is also a clinical social worker, the program identified caregivers of Veterans with dementia who reported experiencing stress and burnout while caring for Veterans with dementia. The DCC provided support via telephone and VVC and also shared resources focused on stress reduction, relaxation techniques, and coping with grief. During 2021, 23 caregivers of diverse racial and ethnic backgrounds were enrolled in the program and 17 were found to have significant caregiver burden. Over 100 support calls were made on the topics of stress reduction, problem solving and community resources. Common problems reported by caregivers included sundowning, sleep problems, grief, and increased social isolation due to the COVID-19 pandemic. Caregivers showed a willingness to participate in the program, indicating that the information, resources and support provided by the DCC helped reduce their stress levels and allowed them to provide ongoing care. Recent program developments, findings from an IRB approved survey of participants, and expansion plans will be reported.

